# A comparative analysis of transcription factor binding models learned from PBM, HT-SELEX and ChIP data

**DOI:** 10.1093/nar/gku117

**Published:** 2014-02-05

**Authors:** Yaron Orenstein, Ron Shamir

**Affiliations:** Blavatnik School of Computer Science, Tel-Aviv University, Tel Aviv 69978, Israel

## Abstract

Understanding gene regulation is a key challenge in today's biology. The new technologies of protein-binding microarrays (PBMs) and high-throughput SELEX (HT-SELEX) allow measurement of the binding intensities of one transcription factor (TF) to numerous synthetic double-stranded DNA sequences in a single experiment. Recently, Jolma *et al.* reported the results of 547 HT-SELEX experiments covering human and mouse TFs. Because 162 of these TFs were also covered by PBM technology, for the first time, a large-scale comparison between implementations of these two *in vitro* technologies is possible. Here we assessed the similarities and differences between binding models, represented as position weight matrices, inferred from PBM and HT-SELEX, and also measured how well these models predict *in vivo* binding. Our results show that HT-SELEX- and PBM-derived models agree for most TFs. For some TFs, the HT-SELEX-derived models are longer versions of the PBM-derived models, whereas for other TFs, the HT-SELEX models match the secondary PBM-derived models. Remarkably, PBM-based 8-mer ranking is more accurate than that of HT-SELEX, but models derived from HT-SELEX predict *in vivo* binding better. In addition, we reveal several biases in HT-SELEX data including nucleotide frequency bias, enrichment of C-rich k-mers and oligos and underrepresentation of palindromes.

## INTRODUCTION

The questions of how, when and where genes are expressed have been fundamental in the field of cell research in the past decades. Transcription factors (TFs) are known to be the main regulators of gene transcription and thus have been a subject for extensive study. These proteins bind to specific short DNA sequence, mainly in the promoter and enhancer regions, and by that impede or encourage transcription. They bind with variable affinity, depending on the sequence and on other factors, and this affinity affects transcription. Learning and modeling the binding preferences of TFs is a central goal in gene regulation research.

Many high-throughput technologies have been developed to study TF binding. Technologies that measure *in vivo* binding include ChIP-chip (1), ChIP-seq (2) and the recently developed ChIP-exo (3). However, measuring *in vivo* binding may not reveal the full picture. First, the accessible sites may not cover the full spectrum of possible DNA k-mers. Second, *in vivo* binding is affected by additional factors, such as chromatin structure, nucleosome positioning and co-factors. As opposed to *in vivo* binding, *in vitro* binding is purely because of direct TF–DNA interaction (or cooperative binding of specific factors) and allows sampling of the full spectrum of DNA k-mers. Technologies that measure *in vitro* binding include protein-binding microarray (PBM) (4) and mechanically induced trapping of molecular interactions (5), both of which measure the binding of a specific protein to a set of oligo sequences designed to cover all k-mers. A newer technology is high-throughput SELEX (HT-SELEX), which consists of several cycles of incubating the DNA-binding protein with a mixture of DNA sequences, enrichment of the bound DNA sequences, sequencing a sample of them and feeding them to the next cycle (6–8).

PBMs have gained great popularity, thanks to their high-throughput and unbiased nature. The public database UniPROBE contains experiments of >400 TFs (9). Although the models derived from this technology have been used extensively, it is unclear how accurate these models are in predicting *in vivo* binding. Several studies have shown that using these positional weight matrix (PWM) models to predict *in vivo* binding leads to poorer results compared with *in vitro* binding prediction (10,11). This performance gap can be explained by several reasons related to *in vivo* binding, such as indirect binding and inaccessibility of genomic DNA. Another possible explanation is that these models include PBM-specific biases. Thus, an independent *in vitro* measurement is required to evaluate the validity of these models.

Recently, a study covering >500 TFs in >800 HT-SELEX experiments was conducted by the Taipale laboratory (12). For the first time, a high number of TFs have available experimental data in two independent *in vitro* technologies: 162 TFs were tested both in HT-SELEX and PBM experiments by the Taipale and Bluyk laboratories, respectively. Jolma *et al.* (12) compared SELEX models with PBM models by length and presented several examples where the SELEX models are more accurate than PBM models based on ChIP-seq data. However, a much broader systematic comparison of the binding models produced by each technology is required.

In this study we aim to analyze and measure the similarities and differences between the two technologies. First, we ask how well HT-SELEX-derived PWM models predict PBM binding. Second, to compare the methods without depending on inferred binding models, we study how well the top k-mers of the two technologies correlate, and which technology is better in k-mer ranking. Third, we test which technology produces better models in predicting *in vivo* binding. Fourth, we uncover biases in HT-SELEX technology. We aim to highlight the advantages of each technology compared with the other. Our observations may help in developing a new method to learn binding models based on HT-SELEX data.

## MATERIALS AND METHODS

### Data

PBM data and PBM-derived PWM models were downloaded from UniPROBE database (9). We used normalized PBM probe data, as available in the database (i.e. the median signal intensity values and corresponding nucleotide probe sequences). Only the 36 bp of unique sequence were used. HT-SELEX experimental data and HT-SELEX-PWM models were downloaded from (12). Human ChIP-seq data were downloaded from ENCODE (13).

### Binding prediction

PWMs were used to represent TF binding preferences (14). For each TF, the set of PWMs reported was used for the binding prediction. In many cases, multiple models were available. In general, we did not distinguish between mouse and human and between the full protein and the binding domain only. For each sequence (either PBM probe or a ChIP-seq peak), the maximum sum occupancy score over the set of PWMs was its predicted binding intensity. For probe sequence s and PWM Θ of length k, the sum occupancy score is

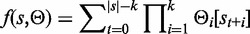

where Θ_i_(x) is the probability of base x in position i of the PWM. A PBM probe is defined as a positive hit for Θ if its binding intensity is greater than the median by at least 4 * (MAD/0.6745), where MAD is the median absolute deviation from the binding intensity median (MAD = 0.6745 for the normal distribution N(0,1)) (15). The positive ChIP-seq peaks are defined as the 500 peaks with the smallest reported *P*-value. We used the 250 bp around the center of the peak as the positive sequence and the 250-bp-long genomic sequence 300 bp downstream of the peak center as the negative sequence. Spearman rank coefficient, sensitivity at 1% false-positive and area under the receiver operating characteristic curve were used to gauge the binding prediction (see (15) for details). For ChIP-seq data, when several experiments were available for the same TF, the average area under curve (AUC) over these experiments is reported.

### Model independent comparison

For each experiment, the scores of the top 100 8-mers according to one technique were compared with their scores in the other technique. PBM 8-mers were scored by average (or median) binding intensity. For a probe *p_i_*_,_ let *s(p_i_)* be its intensity. The score of 8-mer *w* is the average binding intensity: 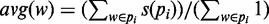.

HT-SELEX 8-mers were scored by either their frequency or ratio of frequencies (frequency in cycle i divided by frequency in cycle i-1). The top 100 8-mers according to their PBM scores were selected, and Pearson correlation was calculated between the PBM scores and the HT-SELEX scores on these 8-mers. Similarly, the top 100 HT-SELEX 8-mers were chosen and their HT-SELEX scores were compared with their PBM scores using Pearson correlation.

### Logo drawing

Motif logos were plotted using http://demo.tinyray.com/weblogo.

## RESULTS

### HT-SELEX-derived models predict PBM binding accurately for most TFs

We first used the HT-SELEX-derived PWM models published in (12) to predict bound probes in PBM experiments and compared their performance with PBM-derived PWM models. We used the SCI09 data set of (16), which includes 115 paired PBM experiments of 104 mouse TFs [in paired experiments, two array designs are used to study the same TF, and so a model learned on one array can be evaluated on the other, see (15)]. For 128 PBM experiments (out of 230), an HT-SELEX-derived model was available for the same TF; this set covers 56 different TFs. For some TFs, Jolma *et al.* reported several PWMs, either because of multiple experiments or because of construction of several PWMs by their algorithm. Occasionally, for a TF analyzed by PBM, both a primary motif and a secondary motif are reported. When multiple PWMs were reported for the same TF by one technology, we assigned to each sequence the highest score obtained by such a model. We used five algorithms to generate PWMs from PBM experiments: Amadeus-PBM (10), Seed-and-Wobble (4), RankMotif++ (15), BEEML-PBM (17) and RAP (18). The performance of the models generated by each algorithm was reported in (18). For each paired experiment, these models were learned on one array and tested on the other to avoid overfitting. Testing of a model was by predicting the binding intensity for each probe in the other array and comparing it with the measured binding intensity. Scores for the comparison were the Spearman rank coefficient on the positive probes, the sensitivity (true positive ratio) at 1% false-positive and AUC of the receiver operating characteristic curve (see Methods). We report the average results in Table 1 (for complete results see Supplementary Table S1).

**Table 1.** Accuracy of HT-SELEX- and PBM-based PWM models in predicting TF binding to PBMs

Model based on	HT-SELEX	PBM				
Algorithm	Jolma *et al.*	Amadeus-PBM	Seed-and-Wobble	RankMotif++	BEEML-PBM	RAP
Spearman rank coefficient	0.282	0.230	0.272	0.301	0.335	0.339
Sensitivity at 1% false-positive	0.288	0.327	0.293	0.277	0.403	0.400
AUC	0.825	0.877	0.872	0.882	0.899	0.898

*Note*. Results show average Spearman rank coefficient, sensitivity at 1% false-positive and AUC for predicting positive binding in 128 paired PBM experiments (covering 56 different TFs). PBM data were taken from (16) and HT-SELEX models were taken from (12). Prediction results for the different PBM-based algorithms were taken from (18). For each experiment the PWM models learned by HT-SELEX or by the other PBM array were used to predict the bound probes (see Methods).

**Figure 1.**
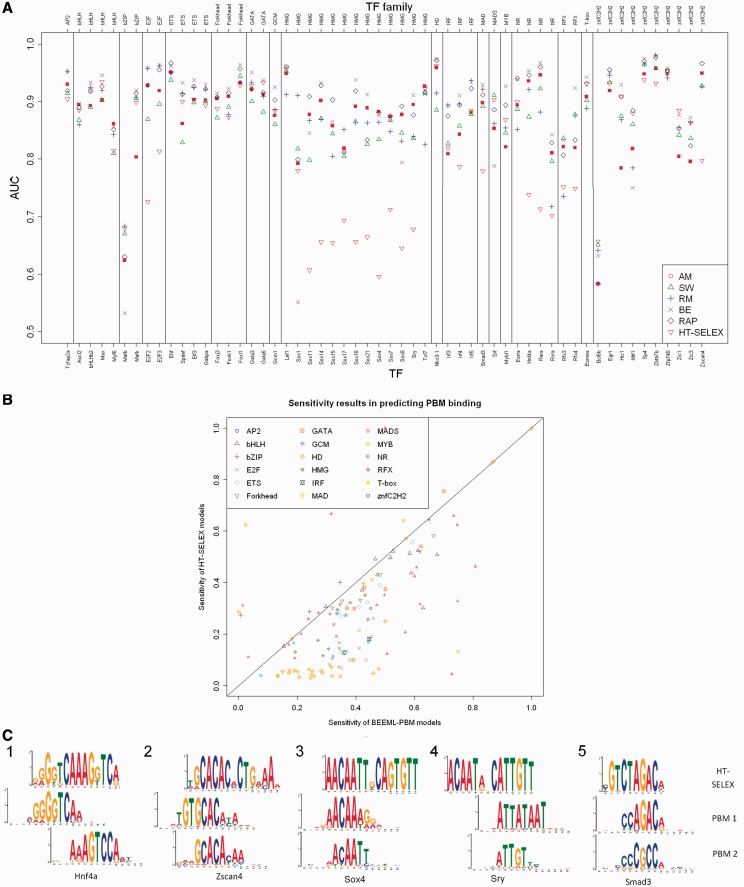
Quality of binding prediction based on PBM data. (**A**) Accuracy in predicting PBM binding. For each PBM experiment, PBM probes are ranked according to motifs inferred by five PBM algorithms (AM = Amadeus-PBM, SW = Seed-and-Wobble, RM = RankMotif++, BE = BEEML-PBM and RAP) and by the HT-SELEX-derived models. This ranking is compared with the true ranking by calculating the AUC for predicting the bound PBM probes. Each dot is the average result of one algorithm in two or four experiments (TF names are listed at the bottom, TF family names are at the top, as given in Jolma *et al.*). (**B**) Sensitivity results in predicting PBM binding. For each PBM experiment, the bound probes were predicted using BEEML-PBM and HT-SELEX PWM models. The plot shows the sensitivity (true positive rate) at 1% false-positive rate of these predictions. Colors correspond to protein families. (**C**) Disagreement between HT-SELEX- and PBM-derived models. The logos are of the PWMs learned from HT-SELEX (top), and PBM (middle and bottom) taken from Jolma *et al.* and UniPROBE, respectively. The middle and bottom models learned from PBM for each TF are the primary and secondary models, respectively. 1, 2: examples where HT-SELEX produces motifs that are similar to the primary PBM model, but too long for PBM technology; 3, 4: cases where HT-SELEX models agree with PBM secondary model; 5: an example where the HT-SELEX model disagrees with both PBM models. (TCF3 was excluded from the analysis because each technology tested a different TF with that name: a bHLH Tcf3 was tested by HT-SELEX, whereas the HMG Tcf3 was tested by PBM).

The results show good agreement between the two technologies (Table 1 and Figure 1A). The average accuracy of HT-SELEX models is significantly lower than that obtained by PBM-derived models (e.g. AUC of 0.825 compared with 0.899 for the best PBM-derived models, *P*-value = 7.68·10^−^^14^ Wilcoxon signed-rank test). This is expected because the evaluation is using PBM measurements. In an additional test on two other PBM data sets covering 115 human and mouse E26 transformation-specific (ETS) and homeodomain TFs tested on a single array (19,20), HT-SELEX-derived models achieved an average AUC of 0.928 (see Supplementary Information). These results may reflect properties of specific TF families.

We found no significant difference between binding models based on mouse and human proteins and between models based on full proteins and binding domains; in both cases the two models performed essentially equally in predicting PBM binding that used mouse binding domains (see Supplementary Information). Note that sample sizes were small and broader tests are still needed.

For some TFs, the HT-SELEX prediction results were poorer than those achieved by PBM models. We define a set of HT-SELEX-derived models for the same TF as a failure if it achieved an AUC lower by at least 0.1 than the average of the five PBM models. HT-SELEX models failed in 20 TFs (covered by 42 experiments), including all Sox, E2F and Rfx proteins, as well as the individual TFs Hnf4a, Rara, Rxra, Smad3, Sry and Zscan4 (Figure 1A and B). These failures occur in particular TF families, including the E2F, Sox, NR, Rfx, MAD and znfC2H2 families [experiments on HMG and znfC2H2 proteins had a low success rate (12)]. The high-mobility group (HMG) super-family includes the Sox, Lef and Tcf protein families. It was suggested that for this family of proteins the DNA structure plays a larger role for binding site recognition than sequence specificity (21), which may explain the failure for this protein family. The recent observation that E2F1 and Smad1 ChIP-seq peaks do not contain the *in vitro* binding site (22) may explain the failures for E2F and Smad3. Figure 1C presents the differences in the models for some of these cases.

### A model-independent comparison

**Figure 2.**
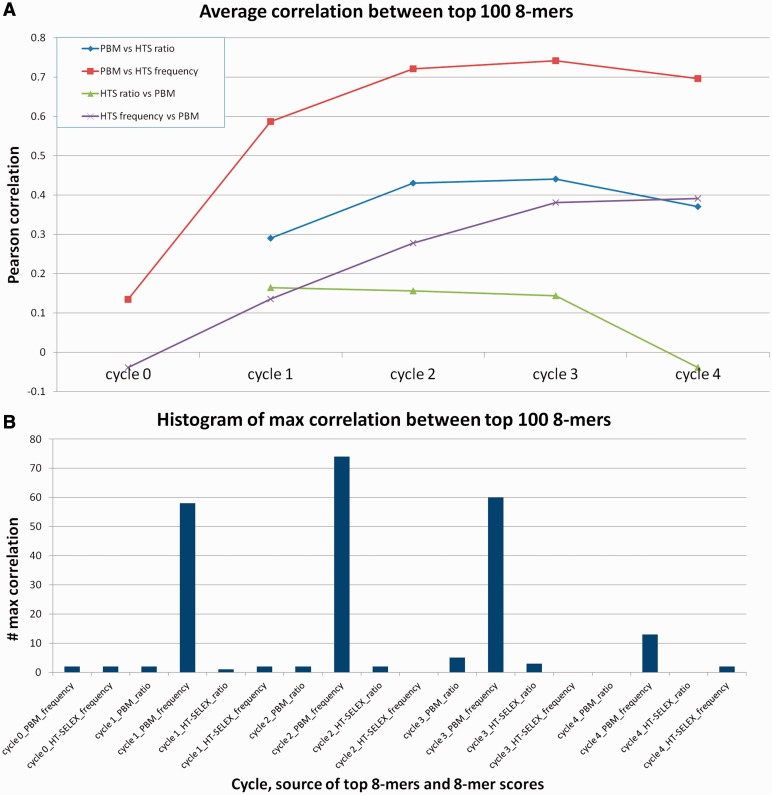
Correlation between the top 8-mers as ranked by PBM and HT-SELEX data. For each HT-SELEX experiment 8-mers were scored by frequency or by the ratio of the frequency to the frequency in the previous cycle. The 8-mers of a PBM experiment on the same TF were scored by average binding intensity. For the 100 top scoring 8-mers according to PBM, the correlation between their PBM scores and their HT-SELEX frequency and ratio scores was computed. Similarly, for the 100 top scoring 8-mers according to HT-SELEX frequency (ratio), their correlation with the PBM scores was computed. (**A**) Average correlation in each cycle. Bar names indicate the technology used to determine the top 100 8-mers. The plot is based on average correlation over 238 TFs. (**B**) Distribution of the maximum correlation for different parameter combinations. The plot shows the number of times the maximum correlation is achieved by each combination of cycle, source of top 8-mers and HT-SELEX 8-mers score. (Because only 39 HT-SELEX experiments included data for a fifth cycle, we excluded it from the comparison; none of these experiments had maximum score at the fifth cycle).

To avoid dependency on model learning, we performed a model-independent comparison. For each HT-SELEX experiment, we selected one arbitrary PBM experiment of the same TF from Cell08, SCI09 or EMBO10 studies. This resulted in 238 PBM-SELEX data sets. We chose to summarize the measurements of each method using 8-mer statistics, and focus on the top ranking 8-mers, which are expected to contain most of the information relevant for TF binding. For PBM 8-mer scores, we used average binding intensity, which is an accurate estimate of binding affinities (18). For HT-SELEX 8-mer scores, we tested two options: 8-mer frequency and 8-mer ratio (frequency in cycle i divided by frequency in cycle i-1) for all cycles (see Methods). With these scores at hand, for each data set we used the set of top 100 8-mers, according to one technology, and calculated the Pearson correlation of its scores with the scores of the same set in the other. Figure 2 shows the results for the different cycles, different scores and different selection of top 8-mers. Complete results are available in Supplementary Table S2. Using the Spearman rank correlation provided similar results (data not shown).

The results show that frequency scores give consistently better correlation with PBM scores than ratios. Hence, for the data analyzed in this study, frequency is superior to ratio, and we used it henceforth. The highest average correlation (just over 0.74) is achieved at cycle 3, when the top 8-mers are selected by PBM data, and HT-SELEX 8-mers are ranked by frequency (Figure 2A). The k-mer ranking becomes more specific as the cycles progress [as was noted in (12)]. At some point it becomes too specific, overrepresenting a small number of top k-mers and thus less accurate for medium- and low-affinity k-mers; we refer to this phenomenon as overspecification. Figure 2B shows, for each combination of cycle, source of top 8-mers and HT-SELEX 8-mer score and the number of times the maximum correlation is achieved by that combination. Cycles 1, 2 and 3 have the highest numbers, supporting the idea of a trade-off between specificity and variability.

The results also suggest that 8-mers ranking using PBM is more reliable than using HT-SELEX. The top 100 PBM 8-mers have greater correlation than the top 100 HT-SELEX 8-mers. Identification of these 8-mers is important for learning the binding preference of the protein. At the current read coverage of HT-SELEX experiments, PBM data are more robust in identifying the top 8-mers. Sequencing a larger sample of the bound oligos may improve 8-mer scores and thus affect the binding models derived from them.

No significant differences were observed when comparing mouse versus human models as well as full protein versus binding domains (see Supplementary Information). Using median binding intensity to score PBM 8-mers instead of the average showed similar results (data not shown).

### HT-SELEX models predict *in vivo* binding more accurately than PBM models

We compared the performance of PBM PWM models with HT-SELEX PWM models in predicting *in vivo* binding. We used human ChIP-seq data from the ENCODE project (13) for TFs that had both PBM and HT-SELEX data. In total, 15 human TFs covered by 111 ChIP-seq experiments were included in this comparison. The top 500 peaks in each experiment were used as a positive set, taking for each peak 250 bp around its center. The negative set consisted of 250-bp-long sequences taken from flanking sequences 300 bp downstream of each positive sequence. This choice is aimed to select negative sequences with statistical features, such as GC-content and k-mer counts, similar to those of the positive ones (23). PBM and HT-SELEX PWM models were taken from UniPROBE database (9) and Jolma *et al.* (12), respectively. When multiple models were reported by one technology, we assigned to each genomic sequence the highest score obtained by such a model. We did not distinguish between human and mouse TFs because Jolma *et al.* (12) reported conservation of binding specificities between these species. Average AUC over the set of ChIP-seq experiments for each TF is reported. Complete results are shown in Supplementary Table S3.

**Figure 3.**
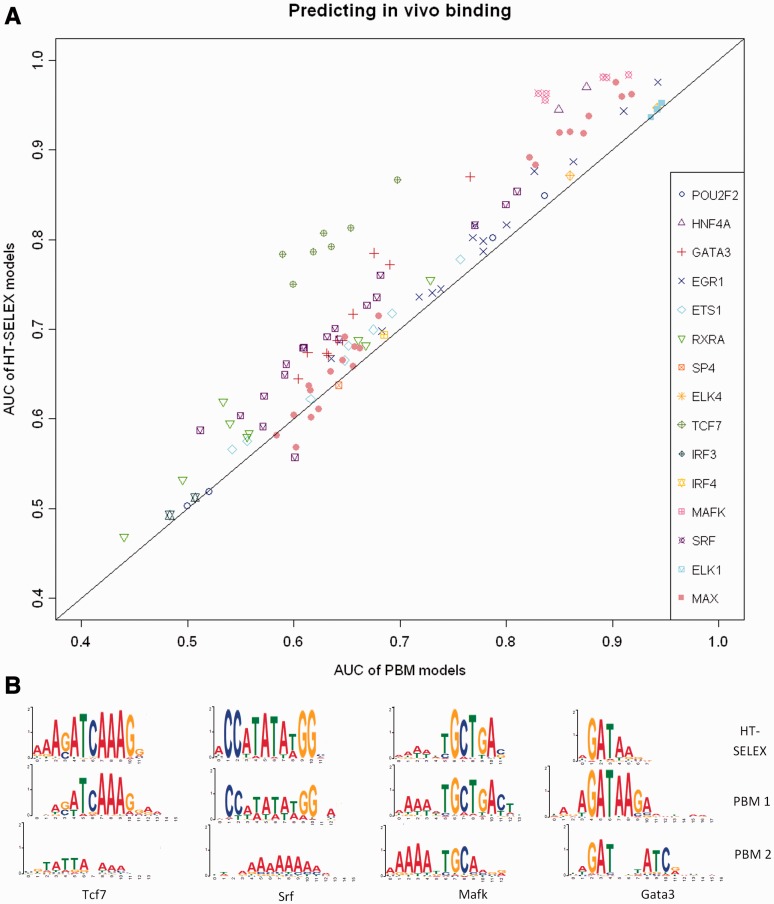
Predicting *in vivo* binding using HT-SELEX- and PBM-derived PWM models. The PWMs learned from HT-SELEX and PBM were taken from Jolma *et al.* and UniPROBE, respectively. *In vivo* binding was measured by the ENCODE project using ChIP-seq. (**A**) AUC results for each ChIP-seq experiment for which HT-SELEX and PBM experiments on the same TF are available. (**B**) Examples where HT-SELEX predicts *in vivo* binding better. For all these examples, the average AUC achieved by the HT-SELEX models exceeds that of the PBM models by >0.05.

Our results show that HT-SELEX models are more accurate in predicting *in vivo* binding (average AUC of 0.756 compared with 0.715, *P*-value = 9·10^−^^5^ Wilcoxon signed-rank test) (Figure 3A). Trimming the PWM to the eight most informative positions results in average AUC of 0.732 and 0.719 (*P*-value = 0.18 Wilcoxon signed-rank test), respectively, hinting that the advantage of HT-SELEX models may be due to the addition of flanking positions. We note that the test set is too small to draw definitive conclusions, but we believe it points to an advantage of HT-SELEX models in predicting *in vivo* binding. For Tcf7, Srf, Mafk, Gata3 and Hnf4a HT-SELEX models, AUC is greater than that of PBM models by > 0.05 (Figure 1C and 3B). When excluding secondary PBM models, for Tcf7 and Mafk the average AUC increased from 0.61 to 0.81 and from 0.87 to 0.92, respectively, suggesting that some secondary models are wrong. At the same time, for Hnf4a the AUC dropped from 0.86 to 0.65. Similar results were observed on mouse ChIP-seq experiments downloaded from the ENCODE project (data not shown). Using the upstream sequences as control gave similar results (data not shown). When using a larger set of 1000 peaks, the advantage of HT-SELEX was smaller but still significant (data not shown).

We checked the effect of the source organism on predicting *in vivo* binding in human. Similarly, we compared the prediction quality based on experiments with full proteins compared to experiments using only the TF binding domains. None of the comparisons showed a significant difference (see Supplementary Information).

### HT-SELEX experiments show systematic biases

**Figure 4.**
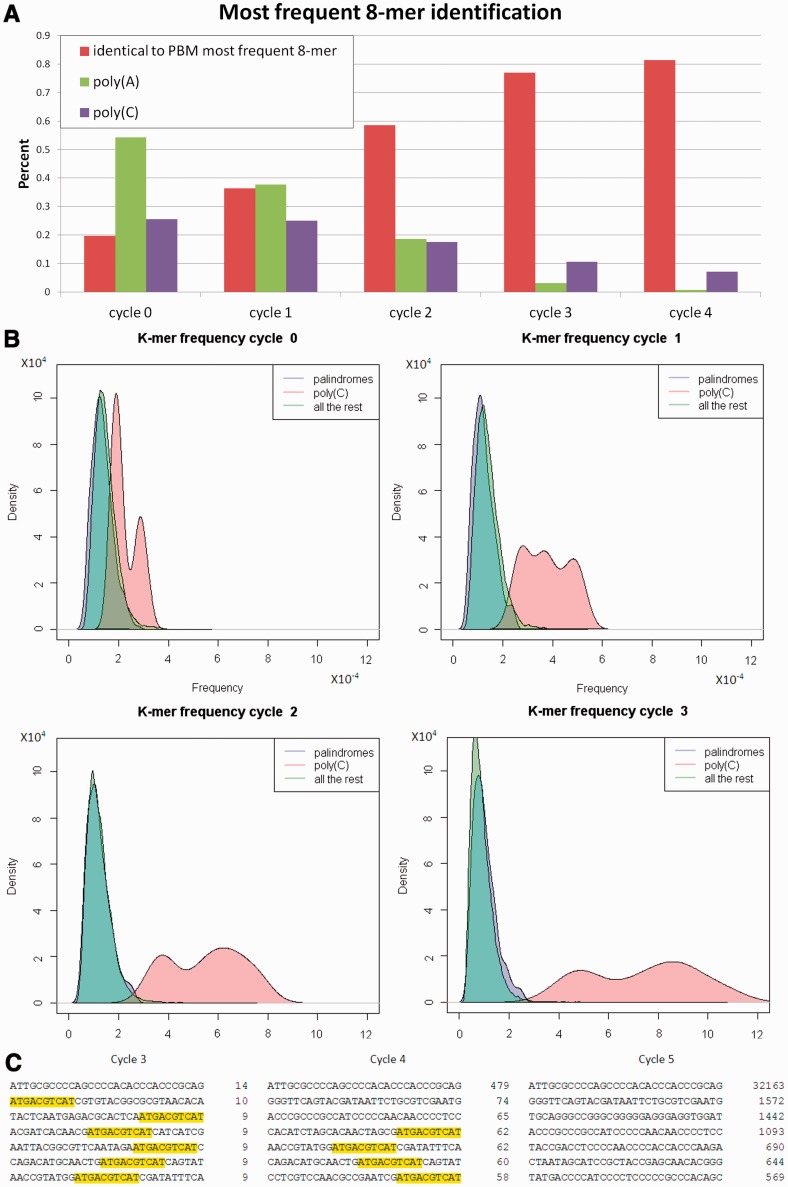
Systematic biases in HT-SELEX technology. (**A**) Properties of the most frequent 8-mer in different cycles. For each cycle, the fraction of times the most frequent 8-mer in the HT-SELEX experiment was poly(A), poly(C) or matched the most frequent 8-mer computed from PBM data is presented (see text). (**B**) The 8-mer frequency density plots for each cycle. The 8-mers were partitioned into three categories: palindromes, poly(C) and all the rest. For each category, a smoothed density plot of its 8-mer frequencies is shown. (**C**) Abundant false oligos in Atf7 HT-SELEX experiment. For cycles 3, 4 and 5, the seven most frequent oligos are shown along with their counts. The consensus sequence is highlighted in yellow (none of the top seven oligos in cycle 5 contain the consensus).

Binding models for HT-SELEX use the most frequent k-mer in some cycle as a seed (6). To study the performance of these models on PBM data, we selected the most frequent 8-mer from each cycle and compared it with the top PBM 8-mer (determined by average binding intensity), when PBM data for the same TF were available (see Methods). We define a positive identification if the top 8-mer is identical with up to two mismatches to the top PBM 8-mer allowing an offset of up to two positions between the aligned sequences. The results are summarized in Figure 4A. Notably, in a substantial number of experiments, the most frequent HT-SELEX 8-mer in the last cycles did not match the top PBM 8-mer. Only 184 of 225 (81%) of the top HT-SELEX 8-mers in cycle 4 matched the top PBM 8-mer. Complete results are available in Supplementary Table S5.

Among the most frequent 8-mers in the different cycles, we observed many A-rich and C-rich 8-mers. To quantify this phenomenon, we focused on poly(A) and poly(C) 8-mers, defined as 8-mers containing at least 7 As or 7 Cs, respectively. Figure 4A shows an overabundance of such 8-mers as the most frequent 8-mers, especially in cycles 0–2. When comparing the distributions of poly(A), poly(C) and of other 8-mers in each cycle over all experiments, poly(A) and poly(C) 8-mers are much more abundant in the initial pool than the other 8-mers (median frequency 1.0·10^−^^3^ and 5.66·10^−^^4^ in cycle 0 and 9.4·10^−^^4^ and 9.43·10^−^^4^ in cycle 1, respectively, *P*-value < 3·10^−^^5^ assuming a uniform null 8-mer distribution).

Moreover, certain 8-mers behaved differently in terms of their frequency changes between cycles. The poly(C) 8-mers were magnified from cycle to cycle much more than other 8-mers (Figure 4B). We also tested palindromic 8-mers (i.e. 8-mers that are identical to their reverse complement). We observed that palindromic 8-mers are less frequent initially (*P*-value = 0.002 in cycle 0 assuming a uniform null 8-mer distribution) and are less magnified than the rest of the 8-mers (Figure 4B, *P*-value = 2.2·10^−^^6^ using a K–S test for comparing the rate of change between cycle 3 and cycle 4 of the palindromes with the other non-poly(A) and non-poly(C) 8-mers). Complete results are available in Supplementary Table S6. Ratio-based statistics showed the same phenomenon (data not shown).

Several reasons can explain the uneven abundance and magnification of k-mers. First, it can arise from technological artifacts. PCR biases have been observed and studied (24), and sequence bias is known to exist in high-throughput sequencing technologies, including the technologies used in Jolma *et al.* study (Illumina Genome Analyzer IIX and Hiseq2000) (25). We observed that nucleotide frequencies in the data are far from uniform, which can be explained by biased oligo generation (see Supplementary Information). Note that both the oligo generation and sequencing processes are strand-specific, so the frequencies of A and T (and of G and C) need not be equal. The systematic overrepresentation of specific k-mers has been observed both *in vivo* [in ChIP-seq data (26)] and *in vitro* [in PBMs (27), where it was termed ‘sticky k-mers’]. According to Jiang *et al.*, in PBM the set of sticky k-mers are all A-rich except CCCCGCCC, in partial agreement with our observations on HT-SELEX data. An alternative explanation suggested by a recent theoretical study was that TFs bind non-specifically to homogenous sequences (28). The underrepresentation of palindromes may be due to the formation of secondary structures that hinder PCR of such sequences (See Supplementary Information).

### False oligos are common in HT-SELEX

Because whole reads (oligos) are sequenced and selected by the HT-SELEX technology, we also conducted an analysis of the abundance and magnification properties of oligos. For each TF, we identified the most frequent oligos in the last cycles. For the 100 most frequent oligos, we defined as false oligos those that do not contain any of the seeds reported in (12) allowing one mismatch. We also measured the oligo enrichment ratio, defined as the oligo’s frequency in the last cycle divided by its frequency in the previous cycle.

The false oligos were on average 25% of the 100 most frequent oligos in the last cycle. In 113 experiments (of 547), at least 50 of the 100 most frequent oligos in the last cycle were false. We observed two characteristics common to them. First, they tended to have more skewed nucleotide distribution than true oligos, with high frequency of one nucleotide (C in 75% of the cases). In all, 35% of the false oligos had one nucleotide composing at least 50% of the sequence, compared with 14% in the true oligos. Second, they tended to be extremely magnified, rising from a low count (or zero) in one cycle to a high count in the next. For example, 41% of the false oligos were not observed in the one-before-last cycle, compared with 19% of the true oligos (note that an oligo present in a particular cycle may have not been observed because of limited sampling). Figure 4C shows an example of Atf7 HT-SELEX experiment. Complete results are available in Supplementary Table S8. Of the previous studies, we observed similar biases in (6) and (8), but not in (7) (see Supplementary Information).

## DISCUSSION

Protein–DNA binding has been in the focus of gene regulation studies for years. In the past, binding sites were defined based on few examples and thus had low resolution and limited accuracy. With technological developments, the ability to measure and predict binding sites has improved. A large leap came in the form of PBMs, which measure *in vitro* the binding intensity of a specific TF to thousands of probes, designed to cover all 10-mers (4). Binding models derived from these data performed well on other PBM data but less so on *in vivo* data (10). One possible explanation was that they reflect PBM artifacts together with the specific binding. How well PBM models represent *in vivo* TF–DNA binding remained an open question.

The emergence of new high-throughput *in vitro* technologies allowed us to deepen our understanding on this question. The HT-SELEX technology measures TF–DNA binding using high-throughput sequencing (6–8). Recently, Jolma *et al.* (12) reported HT-SELEX experiments covering hundreds of TFs, where many of them had been tested on PBM as well. This gave the first opportunity to compare on a large-scale models derived from two independent high-throughput *in vitro* technologies. Through this comparison, we could identify some of the advantages and disadvantages of each technology and determine how relevant *in vitro* models are to *in vivo* binding. A small-scale comparison by Jolma *et al.* (12) covering 14 models reported a few differences.

Our comparison shows that for most TFs the PBM and HT-SELEX technologies produce PWM models that are in good agreement. On average over 246 PBM experiments, the AUC when using the HT-SELEX-derived model for predicting PBM probe binding was 0.875. Moreover, in a model-independent comparison, the average correlation between HT-SELEX 8-mer counts in cycle 3 and PBM average binding intensities over the set of top 100 PBM-ranked 8-mers was 0.74. We observed that PBM-based 8-mer ranking is more accurate and robust than HT-SELEX-based ranking, and that the ranking 8-mers by their occurrence frequency in the Jolma *et al.* HT-SELEX data is better than ranking by between-cycle ratio score. We speculate that this is due to the relatively low read coverage in these experiments [compared with SELEX-seq data, where ratio-based scores were used (7)]. Although each HT-SELEX experiment reported hundreds of thousands of oligos, the SELEX-seq experiments had millions. We conclude that high coverage is necessary to derive accurate ratio scores. For some families of TFs, the two technologies give discordant results, perhaps because of differences in DNA structure [e.g. the HMG proteins, for which structure plays a larger role in binding (21)]. In comparison with *in vivo* data from ChIP-seq experiments, HT-SELEX models had better binding prediction, partly because of the ability to model the side positions more accurately. However, the set of TFs for which HT-SELEX, PBM and ChIP-seq data were available was rather modest, and larger tests are needed.

In analyzing the similarity between the top 8-mers determined by PBM and by HT-SELEX in each cycle, we observed the previously reported phenomenon of overspecification. Although 8-mer frequencies in the initial HT-SELEX cycles are too non-specific and similar to the initial pool (i.e. closer to random), the last cycles can, in some cases, be too specific. There is a trade-off between better coverage of top k-mers in later cycles, which can improve the binding model accuracy, and overrepresentation of few top k-mers, which can make the model too narrow, disregarding weaker binding motifs. This was noted in (6) and in previous studies using the SELEX technology (29).

In the course of our analysis, we observed and characterized several strong biases in many experiments in the HT-SELEX technology. First, we found a systematic bias toward certain types of k-mers [similar but not identical to the ‘sticky k-mers’, reported for PBM data (27)]. For many TFs, in the last cycle C-rich 8-mers are among the most frequent (Figure 4). For example, in 7% of the experiments the most frequent 8-mer in the last cycle contained at least 7 Cs. These phenomena can be explained by PCR and sequencing biases (25) or perhaps by non-specific TF binding (28). Moreover, when measuring oligo (whole read) frequencies, we found that in some experiments the oligos with the highest frequency and those whose frequencies increased fastest between cycles did not contain the binding site; we call them ‘false oligos’. We observed these phenomena in the previous studies (6) and (8), but not in (7). Slattery *et al.* were the only ones to isolate bound oligos through a mobility shift assay, which suggests that this phase removes false oligos and thus improves the quality of the data.

Our analysis suggests that each of the HT-SELEX and PBM technologies has its advantages. PBM data are more accurate and robust in 8-mer ranking; HT-SELEX seems to be superior in *in vivo* binding prediction and allows better learning of longer motifs. We recommend using higher read coverage in HT-SELEX experiments, as was done in (7), to produce more sensitive models. We note that our comparisons and conclusions are limited to the specific technological implementations of HT-SELEX and PBM tested, for which the large-scale overlap exists. Unfortunately, we could not compare SELEX-seq and context-genomic PBMs because of fewer data sets.

Our study aimed to provide deeper and broader analysis of the properties of HT-SELEX experiments and to put them in the context of other high-throughput technologies for evaluating TF–DNA binding *in vivo* and *in vitro*. In the future, we plan to extend this work in several directions. First, we intend to use the new insights to design better motif finding algorithms based on HT-SELEX data. Second, we can learn a binding model based on the biomechanical mechanism of TF–DNA binding using regression methods that use k-mer counts [as in (8)]. Third, we plan to learn more complex binding models. More specifically, we plan to incorporate in the models 2-mer features as well as DNA shape features, as was done recently using custom PBM (30), and demonstrated using existing motif databases (31). The rich and broadly available HT-SELEX data provide a great opportunity to improve our understanding of TF–DNA binding.

## SUPPLEMENTARY DATA

Supplementary Data are available at NAR Online.

## FUNDING

Israel Science Foundation (ISF) [802/08, 317/13]; Edmond J. Safra Center for Bioinformatics at Tel Aviv University, the Dan David Foundation, and the Israeli Center for Research Excellence (I-CORE), Gene Regulation in Complex Human Disease, center 41/11 (to Y.O). Funding for Open Access charge: ISF grant [317/13] and I-CORE.

*Conflict of interest statement*. None declared.

## Supplementary Material

Supplementary Data
