# Supplementary material for: A comparative analysis of transcription factor binding models learned from PBM, HT-SELEX and ChIP data
Source: Nucleic Acids Res. 2014 Feb 5;42(8):e63. doi: 10.1093/nar/gku117 (PMC4005680; doi:10.1093/nar/gku117)
Supplement: Supplementary Data [file supp_42_8_e63__index.html]

A comparative analysis of transcription factor binding models learned from PBM, HT-SELEX and ChIP data — A comparative analysis of transcription factor binding models learned from PBM, HT-SELEX and ChIP data — Supplementary Data 

# A comparative analysis of transcription factor binding models learned from PBM, HT-SELEX and ChIP data

## Supplementary Data

files

**Files in this Data Supplement:**

- Supplementary Data - docx file
- Supplementary Data - xls file
- Supplementary Data - xls file
