# Supplementary material for: A comparative analysis of transcription factor binding models learned from PBM, HT-SELEX and ChIP data
Source: Nucleic Acids Res. 2014 Feb 5;42(8):e63. doi: 10.1093/nar/gku117 (PMC4005680; doi:10.1093/nar/gku117)

**SUPPLEMETARY INFORMTION**

**Predicting PBM binding using HT-SELEX derived models**

Two other PBM studies explored mouse and human TFs: Cell08 ([19](#_ENREF_19)) and EMBO10 ([20](#_ENREF_20)), respectively. In these studies, the experiments were done on a single array design, and thus an unbiased comparison using paired arrays is impossible. Nevertheless, we used the experiments from these studies to gauge HT-SELEX models compared to PBM-derived models. 118 PBM experiments, covering 115 different mouse TFs, had an HT-SELEX model. The average AUC on this set is 0.928 (Tgif1 achieved the minimum AUC: 0.75), higher by 0.1 compared to the SCI09 dataset. The average Spearman rank coefficient and sensitivity at 1% false positive rate are 0.296 and 0.487, respectively. For PBM-derived models we used RAP algorithm in cross-validation. We partitioned the data into two equal sets: one used to learn a model and the other to test it. Average AUC, Spearman rank coefficient and sensitivity at 1% false positive were 0.945, 0.312 and 0.533, respectively. It is expected that PBM-derived models will perform better in predicting PBM binding. Since EMBO10 and Cell08 datasets focused on ETS and Homeodomain proteins, respectively, we attribute the higher HT-SELEX performance on these datasets to the properties of these specific TF families. Together with SCI09 the average AUC is 0.875 (for complete results see Supplementary Table S1).

**Comparing mouse proteins to human proteins and full protein to binding domain models**

We compared the effect of the source organism and of using the binding domain or the full protein on our results. We used for comparison the three benchmarks described in the main text: *in vitro* binding prediction, top 8-mers correlation and *in vivo* binding predicting. All PBM data used in the tests below was of mouse binding domains (since these were the only PBM experiments that had an overlap with proteins measured by HT-SELEX). In all HT-SELEX experiments data were taken from cycle 4.

1. We measured the difference in predicting mouse PBM binding intensities based on mouse and human HT-SELEX. For this aim, we used all proteins for which there were (1) a mouse protein PBM experiment, (2) a mouse protein HT-SELEX experiment, and (3) a human protein HT-SELEX experiment. 51 PBM experiments covering 31 different mouse proteins fit these criteria. The mouse HT-SELEX models achieved average AUC of 0.872, while the human HT-SELEX models achieved 0.869, and the difference was not significant (p=0.1, Wilcoxon signed-rank test).
2. We measured the difference in predicting PBM binding intensities using full proteins and binding domains. We used all proteins that had (a) a binding domain PBM experiment, (b) a binding domain HT-SELEX experiment, and (c) a full protein HT-SELEX experiment. 54 PBM experiments covering 36 different mouse proteins fit these criteria. The full proteins had essentially the same performance as the binding domains (an average AUC of 0.89).
3. We measured the correlation between the top 100 8-mers in human HT-SELEX and mouse PBM, and mouse HT-SELEX and mouse PBM. For this aim, we used the same 31 proteins from A. The human HT-SELEX experiments achieved average correlation of 0.682, while the mouse HT-SELEX experiments achieved 0.712. However, the difference was not significant (p=0.59, Wilcoxon signed-rank test).
4. We used all proteins that had (a) a binding domain PBM experiment and (b) a binding domain HT-SELEX experiment, and (c) a full protein HT-SELEX experiment. We used the same 36 proteins of B for this test. The full proteins had essentially the same performance as binding domains (an average correlation of 0.679 vs. 0.68, respectively).
5. We measured the difference in predicting *in vivo* binding based on mouse HT-SELEX and based on human HT-SELEX. For this aim, we used all human ChIP-seq experiments in ENCODE for which there were a mouse protein HT-SELEX experiment, and a human protein HT-SELEX experiment. 36 ChIP-seq experiments covering 5 different human proteins fit these criteria. The mouse HT-SELEX models achieved average AUC of 0.75, while the human HT-SELEX models achieved 0.76, a non-significant difference (p=0.44, Wilcoxon signed-rank sum test).
6. We measured the difference in predicting *in vivo* binding based on HT-SELEX experiments with full proteins and binding domains. We used all proteins that had (a) a ChIP-seq experiment and (b) a binding domain HT-SELEX experiment, and (c) a full protein HT-SELEX experiment. 124 ChIP-seq experiments covering 13 different human proteins fit these criteria. The binding domains had slightly better performance than the full protein (an average AUC of 0.787 compared to 0.778, respectively, p=0.06 Wilcoxon signed-rank test).

To conclude, we found no significant difference between results based on human and mouse proteins, and no significant difference between results based on full proteins and binding domains. This is in accordance with the conclusions made by Jolma *et al.* Note, however, that larger samples may perhaps reveal significant differences in the future.

**Nucleotide frequency bias in HT-SELEX experiments**

We computed the nucleotide composition of the HT-SELEX sequences to gauge sequence bias. For each experiment, we measured the percentage of A, C, G and T and of the complementary nucleotide pairs. The averages are presented in Figure S1A. There is a clear bias for A and C over G and T and for A+T over G+C. The frequencies of A and C remain roughly constant in all cycles, whereas the frequencies of T and G increase and decrease respectively, with cycle number (see Figure S1A). Interestingly, the frequencies of A and T are different, and so are the frequencies of C and G, indicating a strand bias (This phenomenon has been observed in the Illumina sequencing technology ([32](#_ENREF_32))). Figure S1B shows the A/T percentage per experiment in the initial cycle. The data seem to reflect a mixture of two very distinct distributions. This can be partially explained by the length of the randomly-generated oligos or by batch effects in the initial oligo libraries (see Supplementary Text 4). Complete results in Supplementary Table S4.

**Figure S1**. Nucleotide frequency bias in HT-SELEX experiments. A) Frequency of nucleotides in HT-SELEX each cycle. For each nucleotide and for the complementary nucleotide pairs, their frequency is reported in each cycle. B) Histogram of A/T frequencies over 547 HT-SELEX experiments.


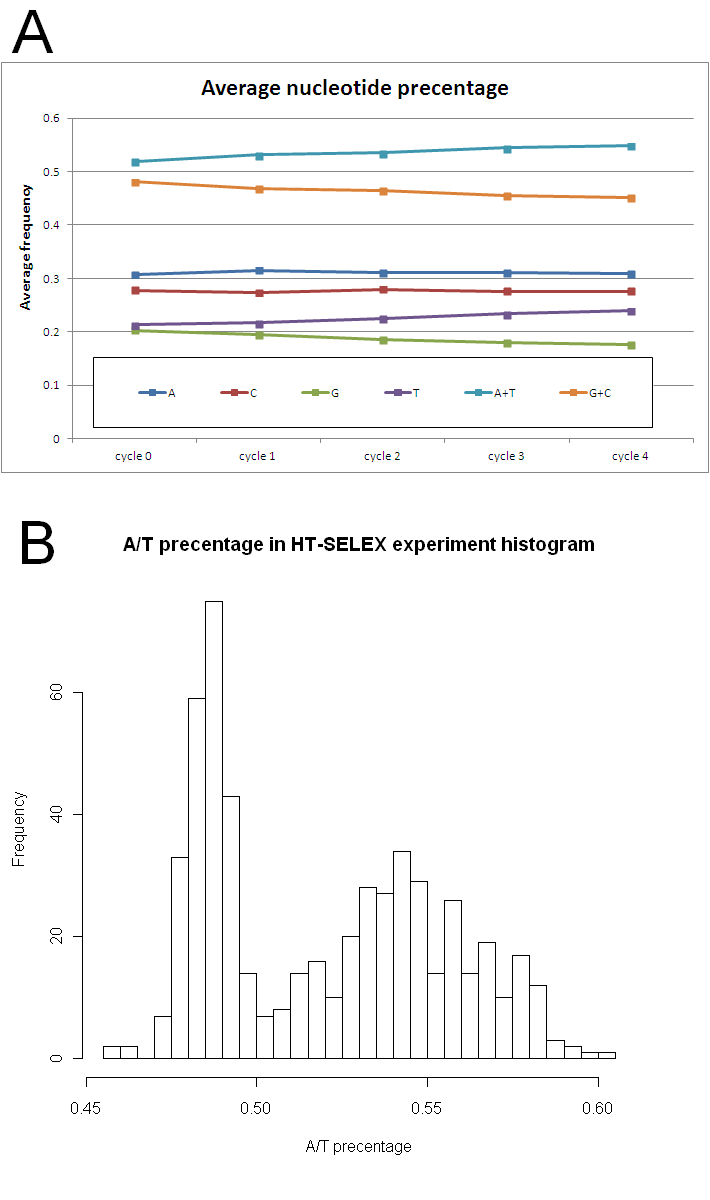


**Is the underrepresentation of palindromes due to secondary structure formation?**

We attributed the lower frequencies of palindromes to their tendency to self-bind by forming a loop-stem structure, thereby reducing their accessibility for the PCR reaction ([33](#_ENREF_33)). To better understand the low frequencies of palindromes, we tested the behavior of gapped palindromes. We define a sequence as a gapped palindrome if it is of the form s-gap-r, where s is a sequence of 4 bases and r is the reverse complement of s, and gap is any sequence of a prescribed length k. We analyzed gapped palindromes with gaps of 0 to 8 bases and calculated their median frequency over all experiments. Figure S2A summarizes the average median frequency over the 256 different palindromes for each gap. Clearly, palindromes with shorter gaps have lower frequency (e.g., p-value=10-5 Wilcoxon signed-rank test, comparing gap 0 to 8 in cycle 4). We speculate that adjacent bases have a higher probability to bond and disturb the PCR process than distant bases. Moreover, a study reported that hairpins with shorter loops have increased stability than longer loops ([34](#_ENREF_34)). We also observed that palindromes with more G-C pairs have lower frequency than those with the same number of A-T pairs, in agreement with the stronger bonding of G-C pairs (see Figure S2B). Palindromes containing A-T pairs only have a higher frequency than random 8-mers, probably due to the sequence bias and the initial A-rich pool. Note that the size of the gap seems to affect median frequency of low G-C palindromes, but not of high G-C palindromes (see Figure S2B). Complete results are available in Supplementary Table S7.

**Figure S2**. Median frequencies of gapped palindromes. A) Average median frequency of gapped palindromes in all HT-SELEX experiments. The average is over all 256 8-long palindromes for each gap (0-8 bases). The average of all other 8-mers is also included for comparison. B) Average median frequency of gapped palindromes in cycle 4 according to GC-content. The frequency profile is shown as a function of the length of the gap (0-8 bases), separated according to GC-content (number of G/C in the first 4 bases). The statistics of all other 8-mers are also included for comparison.

**
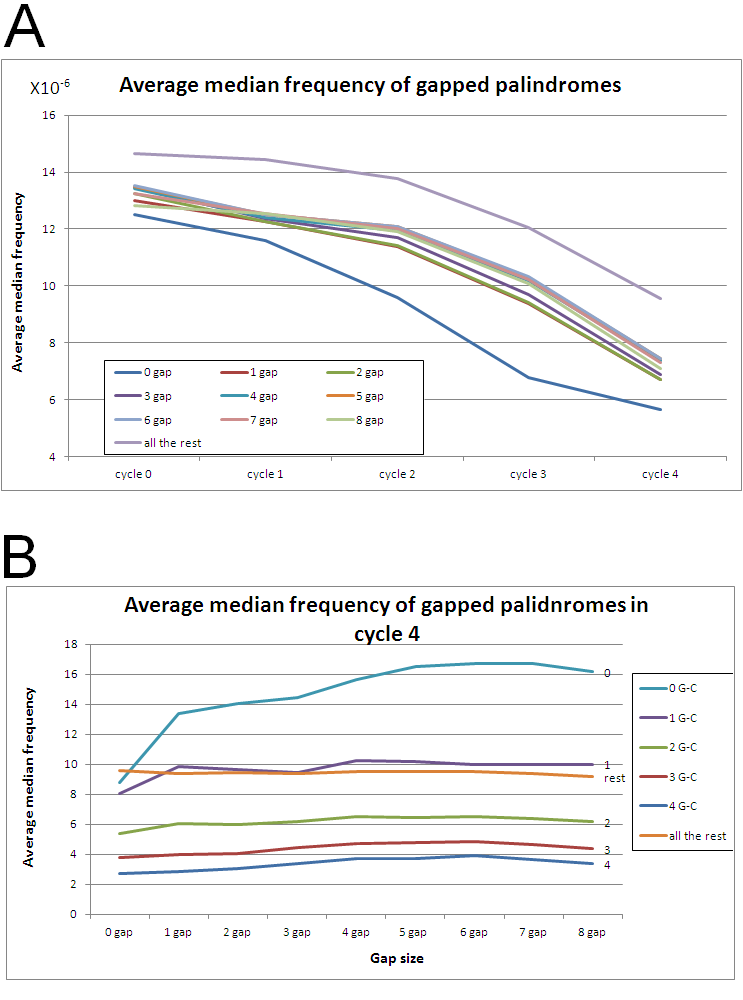
**

**False oligo and nucleotide biases in previous HT-SELEX studies**

We also examined the data from previous studies to see if the phenomena of false oligos and biased nucleotide frequencies are observed in them as well. The first HT-SELEX paper by Zhao *et al*. included one 2-cycle experiment with 10bp oligos on Egr1 protein ([8](#_ENREF_8)). In both cycles, poly(A) and poly(C) are more frequent than expected by chance. Moreover, between the two cycles poly(C) 8-mers are magnified, whereas poly(A) 8-mers decrease in frequency. There were only 11 false oligos in the 100 most frequent oligos. The study of ([6](#_ENREF_6)) reported 19 3-5-cycle experiments with 14bp-oligos. Here too poly(A) and poly(C) were abundant in cycle 0 (median frequency 0.0019 and 0.0013, respectively), and from cycle 1 onwards poly(C) frequency increased while poly(A) decreased. In two of the experiments the number of false oligos was extremely high: 72 and 83 (average of 18 over all experiments). Finally, the 9 available SELEX-seq experiments of ([7](#_ENREF_7)) have much greater coverage (each experiment contains millions of 16bp oligos). Some C-rich oligos exist, but they are in very low numbers. Overrepresentation and between-cycle enrichment of poly(A) and poly(C) 8-mers is not observed in these data. False oligos are almost non-existent. Only two experiments had false oligos (8 and 15 out of the 100 most frequent). This is probably due to the different procedure that was used to clean nonspecific binding: the bound complexes were isolated by electrophoretic mobility shift assays (EMSAs) to ensure that bound oligomers were selected. In conclusion, false oligos, poly(A) and poly(C) biases are observed in previous studies of Jolma *et al*. ([6](#_ENREF_6)) and Zhao *et al*. ([8](#_ENREF_8)), but not in the study of Slattery *et al*. ([7](#_ENREF_7)).

**Nucleotide frequencies vary between experiments due to oligo batch differences**

How can we explain the observation that the frequencies of A+T nucleotides show a bimodal distribution? Since two sequencing platforms were used in the experiments, we tested if the difference can be due to the platforms. We computed the correlation between the nucleotide frequencies in cycle 0 and the sequencing platform used. Correlation to A+T was small (R=-0.08), but higher for G (R=-0.4) and C (R=0.23). All Illumina HiSeq2000 experiments used 30 or 40 base-pairs oligos, and only two of 30 and 40 base-pairs experiments used Illumina Genome Analyzer sequencing, so the oligo lengths and the sequencing platform are highly correlated. Thus, we also computed the correlation to the length of the random part of the oligo, as random generation of oligos is known to be non-uniform in nucleotide composition. The correlation here was much higher for A+T frequency (R=-0.52) and very high for G and T (R=0.94 and R=-0.76, respectively). Another possible source for the biased frequencies is differences in the nucleotide pool between different batches of random oligos produced by the vendor. Indeed, higher correlation was observed between batch number and A+T frequencies (R=0.59) and A and C (R=0.61 and R=-0.68, respectively). Figure S3 shows the distribution of G and T frequencies for different oligo lengths and A+T frequencies for different batches. Thus, we conclude that oligo generation explains to a large extent the different nucleotide frequencies observed in the data.

**Figure S3**. Nucleotide frequencies in HT-SELEX cycle 0. A) Histogram of G frequencies for different oligo lengths. B) Histogram of T frequencies for different oligo lengths. C) Histogram of A+T frequencies for different batches.


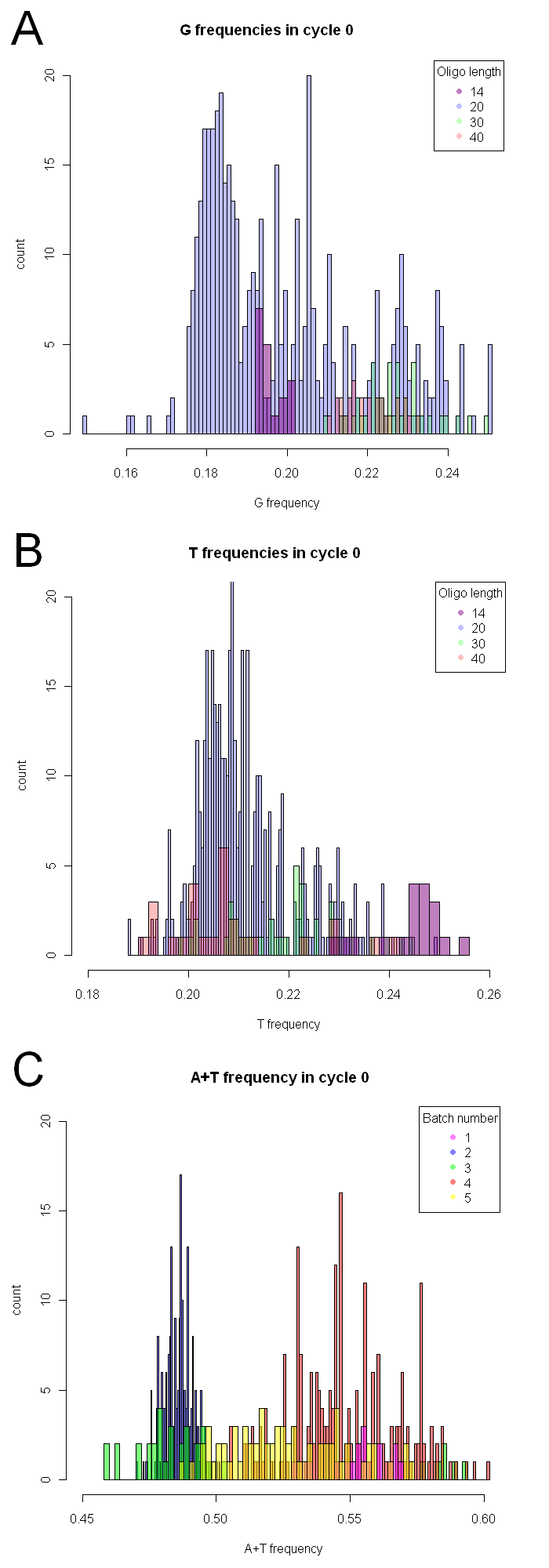

Supplement: Supplementary Data [file supp_gku117_nar-03321-met-k-2013-File006.docx]
